# Maternal Dietary Vitamin D Does Not Program Systemic Inflammation and Bone Health in Adult Female Mice Fed an Obesogenic Diet

**DOI:** 10.3390/nu8110675

**Published:** 2016-10-26

**Authors:** Christopher R. Villa, Jianmin Chen, Bijun Wen, Sandra M. Sacco, Amel Taibi, Wendy E. Ward, Elena M. Comelli

**Affiliations:** 1 Department of Nutritional Sciences, University of Toronto, Toronto, ON M5S 3E2, Canada; christopher.villa@mail.utoronto.ca (C.R.V.); jimmy.chen@utoronto.ca (J.C.); bijun.wen@mail.utoronto.ca (B.W.); amel.taibi@utoronto.ca (A.T.); wward@brocku.ca (W.E.W.); 2 Department of Kinesiology, Brock University, St. Catharines, ON L2S 3A1, Canada; ssacco@brocku.ca; 3 Centre for Child Nutrition and Health, Faculty of Medicine, University of Toronto, Toronto, ON M5S 3E2, Canada

**Keywords:** programming, vitamin D, bone, LPS, gut-bone axis, maternal diet

## Abstract

Obesity is associated with systemic inflammation and impaired bone health. Vitamin D regulates bone metabolism, and has anti-inflammatory properties and epigenetic effects. We showed that exposure to high dietary vitamin D during pregnancy and lactation beneficially programs serum concentration of lipopolysaccharide (LPS) and bone structure in male offspring fed an obesogenic diet. Here we assessed if this effect is also apparent in females. C57BL/6J dams were fed AIN93G diet with high (5000 IU/kg diet) or low (25 IU/kg diet) vitamin D during pregnancy and lactation. Post-weaning, female offspring remained on their respective vitamin D level or were switched and fed a high fat and sucrose diet (44.2% fat, 19.8% sucrose) until age seven months when glucose response, adiposity, serum LPS, and bone mineral, trabecular and cortical structure, and biomechanical strength properties of femur and vertebra were assessed. There was no evidence for a programming effect of vitamin D for any outcomes. However, females exposed to a high vitamin D diet post-weaning had higher bone mineral content (*p* = 0.037) and density (*p* = 0.015) of lumbar vertebra. This post-weaning benefit suggests that in females, bone mineral accrual but not bone structure is compromised with low vitamin D status in utero until weaning in an obesogenic context.

## 1. Introduction

Obesity rates are increasing in both males and females. In 2014, more than 1.9 billion adults (39%) over the age of 18 years were overweight worldwide, with about 600 million (11% of men and 15% of women) classified as obese [1]. This condition is typically associated with a panoply of co-morbidities including impaired bone health [2,3,4]. In fact, while obesity was traditionally considered protective against fragility fracture, more recent data suggest that it is not [5]. Indeed, similar fracture rates are observed among obese and non-obese postmenopausal women but sites of fracture differ, with higher risk of incident ankle and upper leg fractures and lower wrist fracture in obese women [5]. Moreover, sites of fractures differ between the sexes as, for example, obese men were at greater risk for multiple rib fractures compared to men of normal weight [6]. Furthermore, glucose intolerance and type II diabetes that often occurs with obesity are associated with higher risk of fracture, likely due to increased cortical porosity that weakens the structure of bone while bone mineral density (BMD) measurement alone does not accurately predict fracture risk in such individuals [7,8]. Even more disconcerting is the 41 million children under the age of five classified as overweight or obese in 2014 [1]. This is worrisome because many children and adolescents do not outgrow being overweight or obese and tend to continue to gain excess weight in adulthood [9]. In addition, excess weight may affect bone integrity in early life [10], which may dictate the skeletal health in advanced age due to a lower peak bone mass achieved during growth.

Vitamin D is a known regulator of mineral and bone metabolism. Observational studies demonstrate that maternal vitamin D status may be a predictor of offspring bone mineral accrual in females at 9 years of age [11] and up to 20 years of age in both males and females [12]. This may be explained via epigenetic mechanisms including higher methylation of the retinoid-X receptor-alpha (RXRA) cofactor, required for 1,25-dihydroxyvitamin D activation, in offspring born to mothers deficient in vitamin D [13]. This is an example of a concept of early nutrition that invariably impacts later health outcomes and is referred to as nutritional programming. Nutritional programming is used to explain a phenomenon in which a dietary exposure during a critical developmental period results in a permanent or long-term change in the structure or function of an organism [14,15,16]. Given that adiposity negatively regulates bone mineralization [17,18], a combination of both increased adiposity and suboptimal vitamin D status may constitute an inflammatory prone environment, causing poor offspring bone health, which can have lasting implications in adulthood.

Interestingly, we have shown that healthy male mice exposed to low vitamin D in utero and lactation have higher serum lipopolysaccharide (LPS) [19], although females were not studied. LPS is a Gram-negative bacteria-derived inflammatory molecule that may be traced from the diet and the gut microbiota. Circulating LPS affects bone health via increased expression of pro-inflammatory cytokines, including TNF-α and IL-1β, which are known mediators involved in increased osteoclastogenesis [20]. Vitamin D has non-calciotropic capacities to neutralize inflammation [21,22]. In line with this, we have previously shown that exposure to high dietary vitamin D during pregnancy and lactation beneficially programs serum concentrations of LPS, and concomitantly improves trabecular bone structure at both the lumbar vertebra and femur in male offspring fed a high fat and sucrose diet [23]. The purpose of this study was to determine if high dietary vitamin D, fed from conception until weaning, positively programs systemic inflammation and bone health (mineral, structure, strength) in adult female mice receiving the same obesogenic diet.

## 2. Materials and Methods

### 2.1. Animal Study

**Figure 1:**
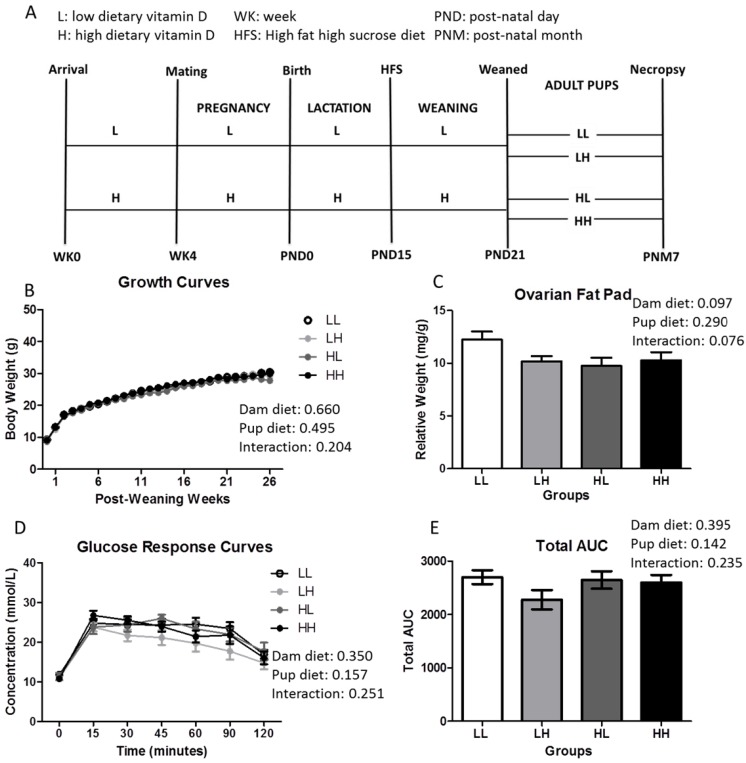
High dietary vitamin D does not affect weight, adiposity, or glycemia independent of greater food intake at seven months of age. (**A**) Study design (*n* = 18–25/group); (**B**) Growth curves (*n* = 18–25); (**C**) Ovarian fat pad weight measured relative to body weight (*n* = 18–25/group); (**D**) Glucose response curves; and (**E**) Total area under the curve following a 2 mg/g bolus of d-glucose oral challenge in mice fasted for 12 h (*n* = 8–12/group). Repeated two-way ANOVA with “dam diet” and “pup diet” as main effects was utilized for growth curve and glucose response curve analysis. Two-way ANOVA with “dam diet” and “pup diet” as main effects was utilized for Total AUC and ovarian fat pad relative weight analysis. Groups are labeled as LL and HH for offspring receiving low vitamin D or high vitamin D from conception until adulthood (25 IU/kg diet or 5000 IU/kg diet, respectively), or switched post-weaning (LH and HL). For significant interactive effects, a one-way ANOVA followed by Tukey’s post-hoc analysis was utilized to determine significance between groups. Values are given as mean ± SEM. Significance between groups set at *p* < 0.05.

The study design (Figure 1A) has been described previously [23] when males were investigated. This study focuses on females obtained through the same breeding. Procedures were approved by the local animal care committee at the University of Toronto (Protocol Number: 20009576). Briefly, three-week-old C57BL/6J mice were purchased from Jackson Laboratories (Bar Harbor, ME, USA) and housed under conventional 12 h:12 h light-dark cycle in a UVB-free incandescent room throughout the study. A standard AIN93G diet containing 1000 IU vitamin D_3_/kg diet was provided to males while females were randomized to an AIN93G diet (Diet # 119290, Dyets Inc., Bethlehem, PA, USA) containing 0.5% calcium with 5000 IU (High, HD) or 25 IU (Low, LD) vitamin D_3_/kg diet ad libitum to attain an optimal (>50 nmol/L) or a deficient (<30 nmol/L) serum vitamin D status, respectively [24]. The amount of dietary vitamin D was independently confirmed by Covance Harlan Teklad (Madison, WI, USA). Pregnant dams were housed individually subsequent to harem style mating. At postnatal day (PND) 15, a high fat and sucrose diet (HFS) (Diet# TD.120612 with low vitamin D (25 IU/kg diet), TD.120613, with high vitamin D (5000 IU/kg diet) Harlan Laboratories, Madison, WI, USA; 44.2% (fat) and 19.8% (sucrose) by kcal, which is approximately double that of the reference AIN93G diet). The protein content of our postnatal diet (19.6%) also closely matches that of the reference AIN93G diet. Micronutrients, except vitamin D, were adjusted proportionately for the higher energy provided in the high fat and sucrose diet to resemble the micronutrient profile in AIN93G diet (Table A1). Female offspring were housed individually at PND 21, and continued on their respective dam’s diet (HH: High, High, *n* = 19; LL: Low, Low, *n* = 23) or were switched to the other (HL: High, Low, *n* = 20; LH: Low, High, *n* = 22) and fed ad libitum until seven months of age. As a result, four treatment groups (HH, LL, HL, and LH) were established, where the first letter denotes the dam’s diet and the second letter denotes the pup’s diet. Weekly measures of food intake and body weight were conducted until seven months of age. At seven months of age, mice were sacrificed by cervical dislocation while blood was collected into LPS-free Pyrotubes (Associates of Cape Code, Falmouth, MA, USA), following cardiac puncture, for endotoxin analysis while another portion was collected into a serum vacutainer. The right femur and the second and third lumbar vertebrae (LV) were cleaned of soft tissue, wrapped in saline soaked gauze, and stored at −80 °C, while organs including the ovarian fat pads, were excised and weighed.

### 2.2. Biochemical Analyses

An enzyme linked immunoassay (ELISA; Cusabio Biotech, Barksdale, DE, USA) was utilized to measure serum LPS concentrations. The Milliplex Map kit (Millipore Corporation, Billerica, MA, USA) was used to assay serum pro-inflammatory cytokines (Tumor necrosis factor alpha, TNF-α; Interleukin-6, IL-6) and bone resorption markers (Sclerostin; Receptor activator nuclear factor kappa-B ligand, RANKL; Osteoprotegerin, OPG) and measured via the Bio-Plex MAGPIX Multiplex Reader (Bio-Rad Laboratories, Hercules, CA, USA). LC-MS/MS was used to determine serum 25(OH)D_3_ concentrations and was performed by the Analytical Facility for Bioactive Molecules of the Centre for the Study of Complex Childhood Diseases, The Hospital for Sick Children (Toronto, ON, Canada).

### 2.3. Glucose Response

Mice were gavaged with 2 mg/g d-glucose following a 12-h fast at seven months of age. A OneTouch Ultra Glucometer (ACCU-CHEK Compact Plus) was used to determine glucose concentrations on 3.5 μL blood collected from the tail vein at baseline (averaged from −30, −15, and 0 min prior to glucose challenge), and at 15, 30, 45, 60, 90, and 120 min post-gavage.

### 2.4. Bone Mineral Content (BMC), Bone Mineral Density (BMD), and Biomechanical Strength of Femurs and Lumbar Vertebrae

Areal BMC and BMD at the whole femur, proximal third of the femur, and LV3 was determined using dual-energy X-ray absorptiometry (DXA) (Orthometrix, White Plains, NY, USA) and a specialized software program (Host Software version 3.9.4; Scanner Software version 1.2.0). Bones were scanned at a speed of 2 mm/s using a resolution of 0.1 × 0.1 mm [25]. To determine biomechanical strength properties at skeletal sites rich in trabecular (i.e., femur neck, LV3) and cortical bone (i.e., femur midpoint), peak load was assessed using a Materials Testing System (Model 4442, Instron Corp., Norwood, MA, USA; Bluehill 2). Femurs were placed on the materials testing machine with the posterior surface of the femur on two 1-mm wide base supports with a jig span width of 6 mm so that the midpoint was positioned directly under the crosshead. The crosshead was lowered at a speed of 2 mm/min until fracture occurred. Peak load was determined as the maximum force the bone can withstand until fracture. To determine peak load at the femur neck, femurs were placed vertically using a customized holder. The crosshead was lowered at a speed of 2 mm/min until fracture at the femur neck occurred. To determine peak load at LV3, bones were placed in the center of a stainless steel plate. A second suspended stainless steel plate was then lowered at a constant rate of 2 mm/min, applying a compressive force to the vertebra until a compression fracture of LV3 was achieved.

### 2.5. Structure of Femurs and Lumbar Vertebrae Using Micro Computed Tomography

Trabecular and cortical bone structural properties of the right femur and second lumbar vertebrae (LV2) were determined using micro-computed tomography (SkyScan 1176, Bruker microCT, Kontich, Belgium) and host software (1176 version 1.1, Bruker microCT, Kontich, Belgium). Individual bones were wrapped in parafilm to retain moisture and then secured axially in a foam holder for scanning. Micro-computed tomography scans were then performed using a 0.25 mm aluminum filter, 9 µm^3^ voxel size, tube voltage of 45 kVp, tube current of 545 uA, 850 ms integration time, and a 0.2° rotation step. All scans were performed over 180° scan with no frame averaging. Scanned images were reconstructed using GPU-accelerated reconstruction (GPUReconServer, Bruker microCT, Kontich, Belguim) and NRecon Reconstruction 64-bit software (Bruker microCT, Kontich, Belgium). Reconstruction parameters for all scanned images included a dynamic image range of 0.000–0.121 of the attenuation coefficient, variable post-alignment compensations, and corrections were applied to smoothing, ring artifacts, beam hardening, and defect pixel masking to reduce signal noise. All scanned images were reconstructed using the same reconstruction parameters and the transaxial images were then saved following reorientation (DataViewer version 1.5.0, Bruker microCT, Kontich, Belgium). Regions of interest (ROI) were then selected by manually drawing contours surrounding trabecular or cortical bone (CTAnalyzer, Bruker microCT, Kontich, Belgium).

#### 2.5.1. Trabecular Bone Structure

ROIs were manually drawn (CTAnalyzer, Bruker microCT, Kontich, Belgium) at two sites rich in trabecular bone to determine site-specific effects of treatment on bone microstructure. At the distal femur metaphysis, the ROI consisted of 0.8795 mm (100 slices) that began 0.528 mm (60 slices) proximally from the metaphyseal side of the growth plate and extended towards the femur head. At the LV, ROIs spanned the length of the LV (0.888–2.709 mm; 101–308 slices) excluding 0.264 mm (30 slices) above and below the cauda and cranial plates, respectively. Saved datasets were then exposed to local thresholding (Distal femur: lower threshold = 75, higher threshold = 255, radius = 8, constant = 0; LV: lower threshold = 82, higher threshold = 255, radius = 8, constant = 0) and a despeckling function to remove white speckles smaller than 10 voxels (CTAnalyzer, Bruker microCT, Kontich, Belgium). The following three-dimensional properties of trabecular bone were then determined: bone volume fraction (BV/TV, %), trabecular thickness (Tb.Th., mm), trabecular number (Tb.N., mm^−1^), and trabecular separation (Tb.Sp., mm).

#### 2.5.2. Cortical Bone Structure

ROIs for cortical bone were manually drawn from the distal end of the third tuberosity extending 0.440 mm (50 slices) towards the ankle. Saved datasets were then exposed to global thresholding (lower threshold = 105, upper threshold = 255) [26] to segment to bone from the background. The following two-dimensional properties of cortical bone were determined: total cross-sectional area inside the periosteal envelope (Tt.Ar., mm^2^), cortical bone area (Ct.Ar., mm^2^), cortical area fraction (Ct.Ar./Tt.Ar., %), and average cortical thickness (Ct.Th., mm).

### 2.6. Statistics

PASW Statistics 18.0 software (SPSS, Inc., Somers, NY, USA) was used to perform all statistical tests, while graphs were generated by GraphPad Prism version 5.01 (GraphPad Software, San Diego, CA, USA). A repeated measures two-way ANOVA was to calculate significant differences in body weight and glucose tolerance curves with “dam diet” and “pup diet” as the main effects, and followed by Tukey’s Honest Significant Difference (HSD) post-hoc analysis for significant interaction effects. All other data were analyzed by two-way ANOVA with “dam diet” and “pup diet” as main factors. Results are presented as means ± SEM. Differences were considered significant at *p* < 0.05.

## 3. Results

### 3.1. High Dietary Vitamin D, Pre- and/or Post-Weaning, Does Not Affect Weight, Adiposity, or Glycemia at Seven Months of Age

**Figure A1:**
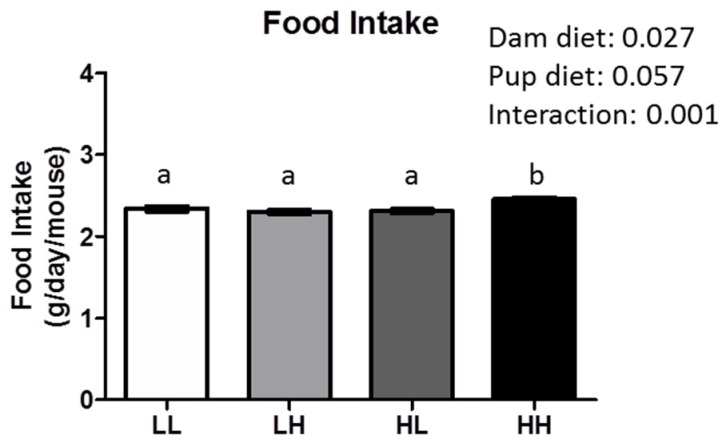
High maternal dietary vitamin D increased food intake in female offspring at 7 months of age. Groups are labeled as LL and HH for offspring receiving low vitamin D or high vitamin D from conception until adulthood (25 IU/kg diet or 5000 IU/kg diet, respectively), or switched post-weaning (LH and HL). Two-way ANOVA was utilized with “dam diet” and “pup diet” as main effects. Values are given as mean ± SEM. Significance between groups set at *p* < 0.05; different letters indicate significance between groups.

Vitamin D status was assessed in one representative mouse per group confirming that concentration of circulating 25(OH)D_3_ is in line with the dietary regimen (LL: 2.10 nmol/L; HL: 0.59 nmol/L; HH: 26.5 nmol/L; LH: 41.9 nmol/L; *n* = 1/group) at seven months of age. Body weight (Figure 1B), ovarian fat pad (OFP) relative weights (Figure 1C), fasting insulin (LL: 212.5 ± 34.0 pmol/L; LH: 206.4 ± 29.2 pmol/L; HL: 154.5 ± 23.9 pmol/L; HH: 153.7 ± 25.4 pmol/L; dam diet: *p*-value = 0.09, pup diet: *p*-value = 0.915, interaction: *p*-value = 0.934), and glycemic response curves and total area under the curve (Figure 1D,E, respectively) were not affected by high dietary vitamin D during in utero and lactation and/or post-weaning until seven months of age, independent of food intake (Figure A1; dam diet: *p* = 0.027, pup diet: *p* = 0.057, interaction: *p* = 0.001).

### 3.2. High Dietary Vitamin D Does Not Program Systemic Inflammation

**Figure 2:**
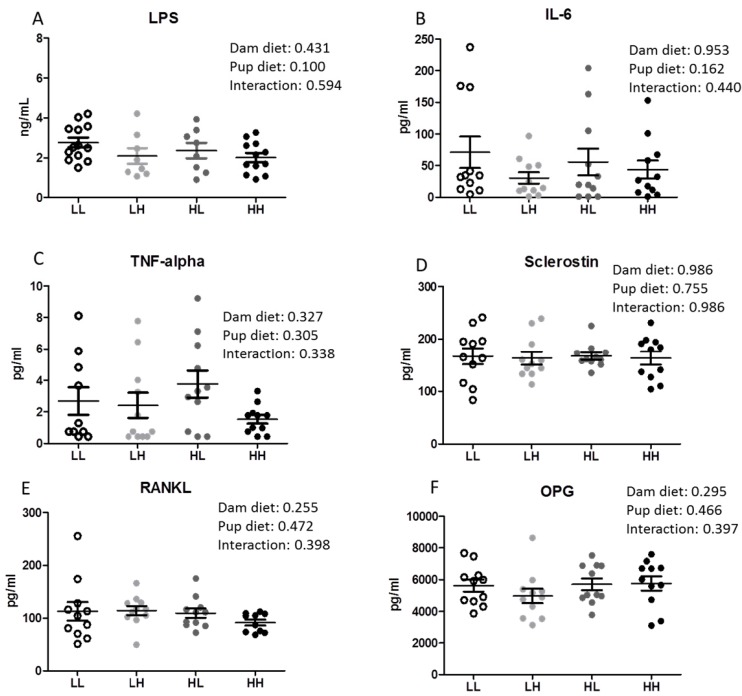
High dietary vitamin D does not reduce serum LPS concentrations in offspring seven months of age. (**A**) Serum LPS concentrations (*n* = 8–13); (**B**) Serum interleukin-6 (IL-6, *n* = 11/group); (**C**) Serum tumor necrosis factor alpha (TNF-α, *n* = 11/group); (**D**) Serum sclerostin (SOST, *n* = 11/group); (**E**) Serum receptor activator nuclear factor kappa-B ligand (RANKL, *n* = 11/group); (**F**) Serum osteoprotegerin (OPG, *n* = 11/group). Groups are labeled as LL and HH for offspring receiving low vitamin D or high vitamin D from conception until adulthood (25 IU/kg diet or 5000 IU/kg diet, respectively), or switched post-weaning (LH and HL). Two-way ANOVA with “dam diet” and “pup diet” as main effects was utilized for results. Values are given as mean ± SEM. Significance between groups set at *p* < 0.05.

There was no effect of high dietary vitamin D consumed either in utero and throughout lactation or post-weaning on systemic inflammation measured as circulating LPS, IL-6, and TNF-α concentrations in pup serum (Figure 2A–C, respectively). In addition, there were no differences in serum markers of bone metabolism (Sclerostin, RANKL, OPG) among groups (Figure 2D–F).

### 3.3. Post-Weaning, but Not Pre-Weaning, Consumption of High Dietary Vitamin D Improves Mineralization in the Lumbar Vertebra but Does Not Increase Strength of Trabecular or Cortical Bone

Mineral accrual of femoral cortical and trabecular bone and the trabecular region of the third lumbar vertebrae (LV3) were analyzed by DXA followed by biomechanical strength testing. Subsequent to weaning, a high dietary vitamin D diet improves both bone mineral content (*p* = 0.037) and density (*p* = 0.015) of LV3 but not of the whole or distal femur. Although higher mineral mass was determined in the LV3, this did not translate into greater strength in this region nor were any differences found in trabecular or cortical femoral regions (Table 1).

### 3.4. High Dietary Vitamin D, Pre- and/or Post-Weaning, Does Not Affect Bone Quality in the Trabecular Bone of the Metaphyseal or the Diaphyseal Region of the Femur

**Figure 3:**
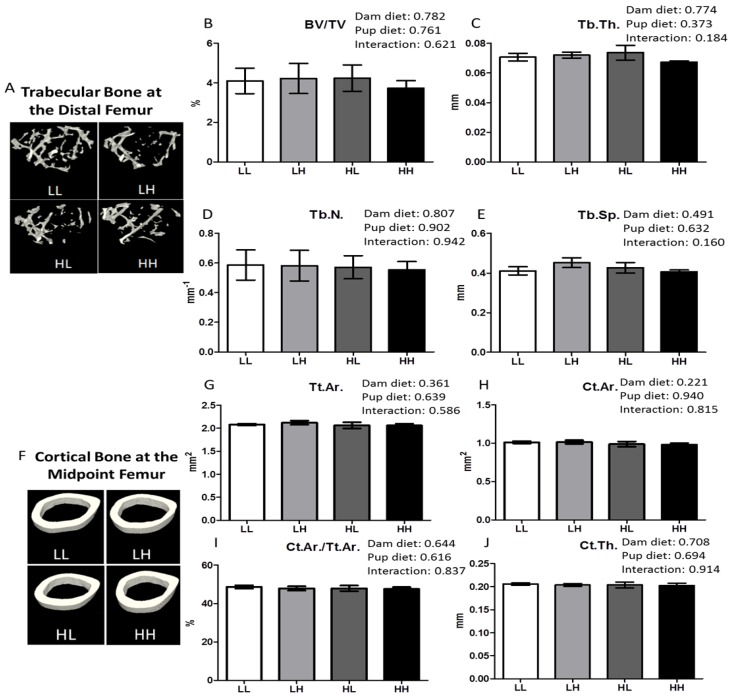
High dietary vitamin D does not affect bone quality in the trabecular bone of the distal femur or the diaphyseal region of the femur. Trabecular bone in the metaphyseal region of the distal femur and the cortical bone of the diaphyseal region of the femur was analyzed using μCT at seven months of age. Representative μCT images of one trabecular section (**A**) and one cortical section (**F**) from each group (LL: Low, Low; LH: Low, High; HL, High, Low; HH, High, High); (**B**) Trabecular bone volume as a percentage of tissue volume, BV/TV%; (**C**) Trabecular thickness, Tb.Th. (mm); (**D**) Trabecular number, Tb.N. (mm^−1^); (**E**) Trabecular separation, Tb.Sp. (mm); (**G**) Total cross-sectional area inside the periosteal envelope, Tt.Ar. (mm^2^); (**H**) Cortical bone area, Ct.Ar. (mm^2^); (**I**) Cortical area fraction, Ct.Ar./Tt.Ar. (%); (**J**) Cortical thickness, Ct.Th. (mm). Values are given as mean ± SEM, *n* = 5–8/group. Two-way ANOVA using “dam diet” and “pup diet” as main effects with significance between groups set at *p* < 0.05.

The qualitative representation of the metaphyseal region of the femur containing trabecular bone is displayed in Figure 3, panel A. Offspring on a high vitamin D diet did not display any benefit in improving trabecular bone in the metaphyseal region of the distal femur in terms of increased bone volume to total volume percentage (Figure 3B, BV/TV%), trabecular thickness (Figure 3C, Tb.Th.), number of trabeculae (Figure 3D, Tb.N.), or decreased trabecular spacing (Figure 3E, Tb.Sp.). In addition, cortical bone quality in the diaphyseal region of the femur midpoint did not qualitatively (Figure 3F) nor quantitatively (Figure 3G–J, *p* > 0.05) differ among groups as a result of high dietary vitamin D consumed until seven months of age.

### 3.5. High Dietary Vitamin D, Pre- and/or Post-Weaning, Does Not Impact Trabecular Bone Quality at the Lumbar Vertebrae

**Figure 4:**
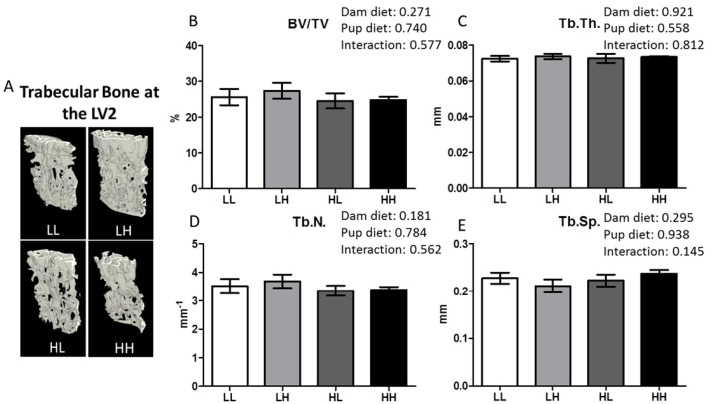
High dietary vitamin D does not impact trabecular bone quality at LV2 as determined using μCT for mice seven months of age. (**A**) Representative μCT images of one trabecular section from each group (LL: Low, Low; LH: Low, High; HL, High, Low; HH, High, High); (**B**) Trabecular bone volume as a percentage of tissue volume, BV/TV%; (**C**) Trabecular thickness, Tb.Th. (mm); (**D**) Trabecular number, Tb.N. (mm^−1^); (**E**) Trabecular separation, Tb.Sp. (mm). Values are given as mean ± SEM, *n* = 6–8/group. Two-way ANOVA using “dam diet” and “pup diet” as main effects with significance between groups set at *p* < 0.05.

The qualitative image of the second lumbar vertebra (LV2) is shown in Figure 4, panel A. High dietary vitamin D did not improve quantitative measures of trabecular bone structure including BV/TV%, Tb.Th., Tb.N., and Tb.Sp. (Figure 4B–E, respectively; *p* > 0.05).

## 4. Discussion

We report that vitamin D deficiency in utero and lactation does not improve metabolic health, particularly systemic inflammation, in adult female mice weaned into an obesogenic environment. Furthermore, there was no improvement in either structure or strength of trabecular or cortical bone in the lumbar vertebra and distal femur. These findings illustrate that vitamin D did not have a nutritional programming effect on any of the parameters measured in these adult female mice. However, there was a non-maternal benefit of consuming high dietary vitamin D on improving bone mineral content and density in the lumbar vertebra, even though this did not translate into better structure and strength. This is in contrast with our recent data obtained in sibling male mice [23]. In sibling males, high dietary vitamin D in utero and lactation had a beneficial programming effect resulting in lower circulating LPS concentration and improved trabecular bone quality in the lumbar vertebrae and distal femur in seven-month-old mice (fed the same high fat and sucrose diet as here). Thus, the in utero and lactation environment were the same for both sexes but outcomes were different. This is an important observation that, if confirmed, may have implications for vitamin D public health strategies targeting our obesogenic environment.

The reason for the lack of observed benefits in females is likely due to vitamin D having sex-specific programming effects, and specifically, that the positive effect of vitamin D is nullified by the obesogenic diet in females but not males. Differences in susceptibility to nutritional programming have been previously observed between the two sexes for metabolic parameters. For example, seven-month-old male rats supplemented with folic acid throughout pregnancy and lactation were 3.7% heavier and had higher fasting blood glucose levels than controls, while females were 5% lighter and did not have different fasting glucose concentrations from controls [27]. Sex specific responses have also been reported for programming of bone health by early postnatal diet in mice. Exposure to soy isoflavones or folic acid during early postnatal life was shown to have a beneficial outcome in terms of higher bone mineral density, improved bone structure and greater bone strength in females but not in males at young adulthood [28,29]. While it was females rather than males that responded, this example nonetheless demonstrates that a food component or nutrient can program bone in a sex-specific manner. Though, to our knowledge ours is the first report of sex differences in response to nutritional programming by vitamin D, particularly considering the bone phenotype, in an obesogenic environment.

Obesity and the metabolic syndrome are alarmingly prevalent worldwide with 22.6% of children and adolescent girls being classified as overweight or obese in 2013 [30] and with the highest prevalence rates of metabolic syndrome (up to 60%) observed in the overweight and obese [31]. Given these statistics, it is imperative that we discern strategies for managing obesity and the morbidities associated with it including bone impairment [5,32]. One such strategy may be early life intervention with vitamin D, which may offer programming benefits that promote health through adulthood. Mechanisms underlying programming effects of vitamin D involve higher methylation of retinoid-X receptor-alpha (RXRA) cofactor in male and female offspring born to mothers deficient in vitamin D [13]. It is not known if sex plays a role in this epigenetic modification of RXRA, but it is known that the global methylation signature is different between newborn boys and girls [33]. At this time, most of the evidence supporting lifelong effects of early life exposure to vitamin D is epidemiological and exemplified by two studies in 9- and 20-year-old females [11,12] suggesting that beneficial programming effects of vitamin D are indeed present in females. Thus, it is likely that these are disrupted by an obesogenic diet. In our study, females are about 33% heavier than same age C57 female mice from Jackson Laboratories fed a regular diet [34]. Moreover, the glycemic responses following an OGTT revealed that serum glucose levels never return to baseline in all groups, indicating that our female mice are diabetic. We know that in healthy males, vitamin D has programming effects [19] and these are maintained in the obesogenic context [23], but studies in females fed a healthful diet are lacking.

Dietary vitamin D, consumed at the same dosage as this study, has a beneficial anti-inflammatory effect as assessed through serum LPS concentration in a healthy model [19]. Circulating LPS, a molecule derived by Gram-negative bacteria residing in the gut, behaves as a stressor for bones and chronic exposure results in impaired trabecular structure at the proximal tibial metaphysis [20]. Opposite to males [23], here vitamin D did not affect serum concentration of LPS resulting in bones being exposed to the same concentration of LPS across the four treatment groups. In line with this, there was no difference in other pro-inflammatory cytokines, bone resorption markers, and either trabecular or cortical bone structure at both sites investigated among the four groups. These findings support the emerging concept of LPS as a mediator of the gut microbiota-bone axis [35]. Remarkably, the lack of difference in structure parameters at seven months of age amongst the four groups was evident despite the post-weaning beneficial effect of vitamin D on BMD and BMC in the lumbar vertebra. In fact, and in line with this, changes to bone mineral content and density are not always accompanied by improved structure or greater strength and therefore, are not an accurate independent predictor of bone health [36,37,38]. Moreover, the post weaning effect is encouraging in that vitamin D supplementation would be a strategy to impact bone mineral accrual in females. This may be particularly important in terms of rescuing deleterious effects of low vitamin D in utero and lactation, given that there are an estimated 20% to 40% of pregnant women who are vitamin D deficient, exposing children to sub-optimal vitamin D during pregnancy [39].

## 5. Conclusions

In conclusion, we found that vitamin D provided from conception until weaning does not program systemic inflammation and bone health in adult females in an obesogenic environment. Vitamin D administration is a nutritional approach of choice to support bone and more recently it has been suggested to also reduce inflammation. Our findings suggest that this strategy may have to be implemented differently in males and females, particularly in the context of an obesogenic (Western) diet.

## Figures and Tables

**Table 1:** Bone mineral and biomechanical strength properties of femurs and LV from female C57BL/6J mice ^a^.

	Dam Diet	Pup Diet		*p*-Value		
		Low	High	Dam Diet	Pup Diet	Dam Diet × Pup Diet
*Bone mineral*						
Whole femur						
BMC, mg	Low	25.5 ± 0.39	25.7 ± 0.36			
	High	26.1 ± 0.51	26.3 ± 0.43	NS	NS	NS
BMD, mg/cm^2^	Low	62.8 ± 0.77	62.7 ± 0.67			
	High	64.2 ± 0.71	63.7 ± 0.86	NS	NS	NS
1/3 Proximal femur						
BMC, mg	Low	9.4 ± 0.20	9.9 ± 0.19			
	High	9.7 ± 0.23	9.8 ± 0.25	NS	NS	NS
BMD, mg/cm^2^	Low	67.9 ± 1.1	68.9 ± 0.85			
	High	68.9 ± 1.2	68.8 ± 1.10	NS	NS	NS
LV3						
BMC, mg	Low	22.3 ± 0.48	22.9 ± 0.82			
	High	21.8 ± 0.60	24.2 ± 0.81	NS	0.037	NS
BMD, mg/cm^2^	Low	60.3 ± 1.02	61.7 ± 0.76			
	High	60.0 ± 0.90	62.5 ± 0.87	NS	0.015	NS
*Biomechanical strength*						
Femur midpoint						
Peak load, *N*	Low	21.9 ± 0.85	21.8 ± 0.83			
	High	20.2 ± 0.53	21.1 ± 0.48	NS	NS	NS
Femur neck						
Peak load, *N*	Low	15.3 ± 0.90	16.6 ± 0.91			
	High	15.1 ± 1.95	14.1 ± 1.23	NS	NS	NS
LV3						
Peak load, *N*	Low	42.8 ± 2.87	41.6 ± 3.60			
	High	37.7 ± 3.60	37.5 ± 3.74	NS	NS	NS

^a^ Values are expressed as mean ± SEM. Vitamin D levels: 25 IU/kg diet (LOW); 5000 IU/kg diet (HIGH). For bone mineral outcomes: *n* = 13–15/group. For biomechanical strength outcomes: *n* = 12–15/group. NS, non-significant, *p* > 0.05; BMC, bone mineral content; BMD, bone mineral density; LV, lumbar vertebrae.

## Appendix A

**Table A1:** Composition and macronutrient distribution of the high fat and sucrose diet containing low or high vitamin D.

**Diet Formula**	**g/kg**
Casein, “Vitamin-Free” Test	245.0
L-Cystine	3.5
Corn Starch	85.0
Maltodextrin	115.0
Sucrose	199.7
Vegetable Shortening, hydrogenated (Primex)	195.0
Soybean Oil	30.0
Cellulose	58.0
Mineral Mix, AIN-93G-MX (94046)	43.0
Calcium Phosphate, dibasic	3.4
Vitamin Mix, AIN-93 w/o A, D, E (120379)	19.0
Vitamin A Palmitate (500,000 IU/g)	0.015
Vitamin D_3_, cholecalciferol (50,000 IU/g in sucrose)	0.1 † or 0.0005 ††
Vitamin E, DL-alpha tocopheryl acetate (500 IU/g)	0.285
Choline Bitartrate	3.0
**Vitamin Mix**	**g/kg**
Niacin	3.0
Calcium Pantothenate	1.6
Pyridoxine HCl	0.7
Thiamine HCl	0.6
Riboflavin	0.6
Folic Acid	0.2
Biotin	0.02
Vitamin B_12_ (0.1% in mannitol)	2.5
Vitamin K_1_, phylloquinone	0.075
Sucrose, fine ground	990.705
**Mineral Mix**	**g/kg**
Calcium Carbonate	357.0
Potassium Phosphate, monobasic	196.0
Potassium Citrate, monohydrate	70.78
Sodium Chloride	74.0
Potassium Sulfate	46.6
Magnesium Oxide	24.3
Ferric Citrate	6.06
Zinc Carbonate	1.65
Manganous Carbonate	0.63
Cupric Carbonate	0.31
Potassium Iodate	0.01
Sodium Selenate	0.0103
Ammonium Paramolybdate, tetrahydrate	0.008
Sodium Meta-Silicate, nonahydrate	1.45
Chromium Potassium Sulfate, dodecahydrate	0.275
Lithium Chloride	0.0174
Boric Acid	0.0815
Sodium Fluoride	0.0635
Nickel Carbonate Hydroxide, tetrahydrate	0.0318
Ammonium Meta-Vanadate	0.0066
Sucrose, fine ground	220.7159
**Macronutrient**	**% kcal**
Protein	19.6
Fat	44.2
Carbohydrate (Sucrose)	36.2 (19.8)

† Vitamin D content of modified high vitamin D AIN93G diet; †† Vitamin D content of modified low vitamin D AIN93G diet.
